# Gold Nanoparticles for Vectorization of Nucleic Acids for Cancer Therapeutics

**DOI:** 10.3390/molecules25153489

**Published:** 2020-07-31

**Authors:** Daniela Ferreira, David Fontinha, Catarina Martins, David Pires, Alexandra R. Fernandes, Pedro V. Baptista

**Affiliations:** UCIBIO, Dept. of Life Sciences, Faculdade de Ciências e Tecnologia, Universidade NOVA de Lisboa, Campus de Caparica, 2829-516 Caparica, Portugal; danielafcferreiraa@gmail.com (D.F.); dj.fontinha@campus.fct.unl.pt (D.F.); cf.martins@campus.fct.unl.pt (C.M.); dfs.pires@campus.fct.unl.pt (D.P.)

**Keywords:** gene editing, gene silencing, gold nanoparticles, nanomedicine, therapeutic nucleic acid

## Abstract

Cancer remains a complex medical challenge and one of the leading causes of death worldwide. Nanomedicines have been proposed as innovative platforms to tackle these complex diseases, where the combination of several treatment strategies might enhance therapy success. Among these nanomedicines, nanoparticle mediated delivery of nucleic acids has been put forward as key instrument to modulate gene expression, be it targeted gene silencing, interference RNA mechanisms and/or gene edition. These novel delivery systems have strongly relied on nanoparticles and, in particular, gold nanoparticles (AuNPs) have paved the way for efficient delivery systems due to the possibility to fine-tune their size, shape and surface properties, coupled to the ease of functionalization with different biomolecules. Herein, we shall address the different molecular tools for modulation of expression of oncogenes and tumor suppressor genes and discuss the state-of-the-art of AuNP functionalization for nucleic acid delivery both in vitro and in vivo models. Furthermore, we shall highlight the clinical applications of these spherical AuNP based conjugates for gene delivery, current challenges, and future perspectives in nanomedicine.

## 1. Introduction

### 1.1. Cancer: Worldwide Statistics; Biomarker Detection and Treatment Strategies

Cancer comprises a set of diseases arising from the uncontrolled growth and proliferation of abnormal cells (tumor), which generally hijack the molecular mechanisms of cell regulation and promote invasion of tissues, alteration of local metabolic and physiologic conditions that may, at later stages and provided conditions exist, be capable of dissemination throughout the whole organism and colonization of different tissues and organs (metastasis). The World Health Organization (WHO) estimates cancer as the second leading cause of death worldwide, resulting in approximately 10 million deaths in 2018 [1]. The onset and progress of a tumor are characterized by a series of molecular deviations, that deregulate canonical pathways of cell development and dramatically alter the constraint imposed by the body on the individual cell. This is perhaps one of the most critical properties of cancer cells—lack of response to cell cycle control. These random molecular alterations influence not only the foundation of the tumor but also the degree of differentiation and invasive capability of cancer cells [2].

Presently, there has been a significant advance towards the identification and characterization of oncological biomarkers, which have put forward new and more efficient approaches for the recognition of cancer cells and offering of tools for the molecular staging of cancer progression. These advances have been crucial for the molecular detection and early diagnosis of cancer, and for the development of new and more precise treatment strategies [3,4,5]. Among the plethora of molecular biomarkers in cancer, those able to pinpoint germline or somatic mutations, transcriptional changes, and post-translational modifications have been at the forefront of contributors to the advance of innovative cancer diagnostics and therapeutics [6].

Still, upon diagnosis, the main treatment options for cancer revolve around surgery, radiation, chemotherapy, immunotherapy, or a combination of these therapies. Despite the undisputed contribution of these approaches, these are invasive techniques and/or usually lack selectivity towards cancer cells, thus contributing to a wide range of deleterious side-effects that often hamper therapy success, causing relapse and a considerable lack of quality of life for patients. As such, there has been an increasing demand for alternative non-invasive and selective methods to overcome these challenges. Some of these more precise approaches often include directing/targeting drugs directly at tumor cells via molecular actuators, antibodies and peptides that promote the destruction of cancer cells whilst sparing the surrounding healthy tissue (e.g., immune drug conjugates, gene therapy, etc.) [7].

### 1.2. Gene Therapy in Cancer

Gene therapy has been an attractive approach against cancer. In fact, the concept of introducing therapeutic nucleic acids (TNAs) into target cells in a controlled manner, with the objective of blocking the expression of specific genes that have been promoting cancer development (gene silencing) or restoring the expression of tumor suppressor genes, which have lost their function during cancer ontogeny, has been extensively researched. Currently, there are a wide range of gene therapy strategies that rely on the capability of vectorizing TNAs and/or a combination of tools for gene edition—that aim at correcting molecular events contributing to cancer development—into the desired target cells. These platforms include gene and genome editing tools, elements capable of inducing and modulating mechanisms of endogenous RNA interference (RNAi), and those able to target and destroy aberrant molecular markers [8,9]. Gene therapy can target DNA, using genome editing tools such as clustered regularly interspaced short palindromic repeats (CRISPR), meganucleases, transcription activator-like effector nucleases (TALENs) and zinc finger nucleases (ZFNs); or RNA, using antisense oligonucleotides (ASOs), RNAi mechanisms, ribozymes and riboswitches [10]. Herein, we shall briefly discuss some of the most promising tools, either conceptual or already translating to the clinics.

#### 1.2.1. Genome Editing Tools

##### CRISPR/Cas9

CRISPRs were revealed in bacteria as part of their adaptive defense against foreign DNA from infecting viruses, phages and plasmids by inducing RNA-guided DNA cleavage [11]. Substantially, CRISPR-Cas systems can be organized in two major classes, considering the structure of the Cas genes and the way they are organized [12,13]. Class 1 CRISPR–Cas systems comprise multiprotein complexes, though class 2 systems encompass one effector protein [14]. The CRISPR/Cas9 system, a subtype of class 2 systems, is the most used [15]. It includes a single-stranded guide RNA (sgRNA), designed to complement and bind to the target DNA site in a sequence-specific manner, followed by a protospacer adjacent motif (PAM) to ensure compatibility with the Cas9 protein, and a Cas9 endonuclease, that precisely cleaves the DNA to generate a double strand break (DSB) [16]. The CRISPR/Cas9 system allows for targeted genomic modifications and there are three main execution strategies: plasmid-based CRISPR/Cas9 strategy; direct delivery of Cas9 mRNA and sgRNA; direct delivery of Cas9 protein and sgRNA56 [17].

##### ZFNs-Zinc Finger Nucleases

ZFNs result from the fusion of a cleavage domain that is not sequence-specific to a site-specific DNA-binding domain—the zinc finger [18]. Each zinc-finger unit recognizes three base pairs within the major groove of DNA [19]. The DNA cleaving domain is formed by a FokI type II restriction endonuclease, which can be dimerized to directly target the desired genomic sequences [20]. The specificity of ZFNs to the target is determined by each finger’s amino acid sequence, the number of fingers, and the interaction of the nuclease domain. Due to the ZFNs’ modular structure, it is possible to optimize both functional domains to enable the development of novel modules for genome editing [21].

##### TALENs-Transcription Activator-Like Effector Nucleases

TALENs encompass a nonspecific DNA cleavage domain united to a custom-made sequence-specific binding domain that produce double strand breaks (DSBs). The DNA-binding domain derives from a highly conserved repeat sequence from transcription activator-like effector (TALE). [22]. A functional FokI endonuclease is responsible for the site-specific DSBs hence stimulating DNA recombination to target genome alteration. Like what happens with ZFNs, the FokI cleavage domain must be dimerized to cut the two strands of the target DNA sequence. As such, TALEN modules are designed in pairs to bind opposing DNA target sequences, including appropriate spacing between the two binding sites [23]. Additional effort has been put into adding multiple effector domains for other modification purposes, including nuclease activity, transcriptional modulators and activators, and site-specific recombinases [24].

Even though cipher codes provide for a simple design when compared to triplet-confined ZFN, the large-scale assembly of matching repeat sequences is still a main limitation for cloning repeat TALE arrays. As such, several strategies have been proposed to overcome these bottlenecks, such as “Golden Gate” cloning [24], high-throughput assembly using solid phases [25] and connection independent cloning [26].

##### Meganucleases

Meganucleases are a specific group of endodeoxyribonucleases characterized by large motifs that target specific nucleotide sequences conferring the capability to replace, eliminate, or modify those sequences of nucleotide. These meganucleases are typically grouped into five families: H-N-H, His-Cys box, GIY-YIG, PD-(D/E)XK and LAGLIDADG. Amongst these, the LAGLIDADG family of homing endonucleases shows a range of growing applications as gene editing tool mainly toward modulation of splicing and self-splicing events, maturation of RNA [27]. Meganucleases may also be used for genome (DNA) edition. For example, DmoCre and E-Drel are proteins variants combined with meganucleases, which provides for an enhanced capability in targeted cleavage. Meganucleases tend to exhibit low cell toxicity, but their future clinical application still requires further studies on the impact to tissues and organs, and the systemic effects to the organism [28].

An overview of the use of gene editing tools in clinical trials is presented in Table 1.

#### 1.2.2. Gene Silencing—RNAi; ASOs; Ribozymes and Riboswitches

Gene silencing consists in the intracellular delivery of TNAs, usually relying on RNAi for modulation of gene expression, such as short hairpin RNA (shRNA), small interfering RNA (siRNA), and microRNA (miRNA). Most gene silencing targets are related to oncogenes (e.g., *cMYC*, *KRAS*, *BCR-ABL*, *EGFR*), which trigger abnormal cell proliferation due to the increase in gene expression or up-activity of the resulting onco-proteins; may also inhibit tumor suppressor genes, that normally prevent cell proliferation and tumor development; or influence other genes related to tumoral survival (e.g., angiogenesis) [29].

##### RNAi Mechanisms and ASOs

RNAi technology has been introduced as a novel targeting therapeutic strategy with particular interest in cancer. miRNA is engaged with the cytoplasmatic RNA-induced silencing complex (RISC), thus activating mRNA degradation or repression of translation. RNAi is mediated by a dsRNA, which is used to regulate gene expression [30]. ASOs (e.g., antisense DNA technology) allow to inhibit or downregulate the production of a particular protein by using antisense DNA molecules complementary to the target sequence in the cell. Antisense DNA is also a valuable tool towards regulation of gene expression, which has already been used in combination with conventional chemotherapy in cancer. The most used in gene silencing is the RNAi technology, more specifically siRNA [31].

The CAR T strategy is based on the ex vivo editing of T cells receptors to specifically target and kill cancer cells. Following edition of the autologous CAR T cells, these are extended to subsequently injected back to the patient. This therapeutic strategy has had reasonable success against liquid tumors (e.g., B lymphomas) and, more recently, to tackle other types of cancer [32,33,34].

##### Ribozymes and Riboswitches

There are catalytic RNAs (ribozymes) that participate in a range of cellular processes in an autonomous manner. Ribozymes catalyze RNA strand hydrolyses and ligation reactions and are usually grouped according to their main function in two major categories: cleaving ribozymes and splicing ribozymes [35]. The later can be used to regulate gene expression through precise mRNA splicing. Riboswitches and other RNA-based sensors may be associated to the regulation of gene expression via modification of RNA conformation, in response to both internal and external triggers, for which they do not require any protein. The term riboswitch is normally applied to “metabolite sensing RNA switches”, capable to respond to ions and other small molecules, temperature, and other regulatory RNAs [35].

These molecular tools to modulate genome and gene expression have been used in several proof-of-concept strategies for tracking tumor progression, and even to kill cancer cells. However, the choice to use one or another is not straightforward since these strategies show some crucial limitations of use. Table 2 highlights some of the key features of these strategies and directs towards some of the most recent applications. Most limitations may be resolved through the design of proper formulation and delivery strategy, which should be capable of transporting high payloads of the desired molecular actuator with high cell precision, thus increasing the circulation half-life and conferring protection against degradation by endogenous mechanisms. As such, strong efforts have been put towards the development of new platforms capable to circumvent these issues. It should be notes that CRISPR-Cas9 technology has been considerably optimized, thus offering more advantages when compared to other. Such optimizations have been directed towards increasing flexibility, cost-effectiveness and convenience [36,37]. CRISPR-Cas9 technology also have the advantage of use for both knock-down and for insertion in genomes (i.e., addition of sequences and editing).

Still, these editing tools may trigger off-target effects, such as erroneous integration, disruption of gene function, which more generally occur due to unintended binding, modification, and cleavage of nucleic acids. Of relevance is also the correct gene editing function but the inefficient targeting to the desired cell or sub-cellular compartment, where nanoparticles have been capable to provide solutions in vectorization [38]. Recent studies show that ribonucleoproteins adsorbed onto AuNPs (termed CRISPR-Gold) were capable to correct the dystrophin gene in a mouse model of Duchenne muscular dystrophy, leading to improved clinical phenotypes following intramuscular injection [39].

## 2. Molecular Nanomedicines against Cancer

**Figure 1:**
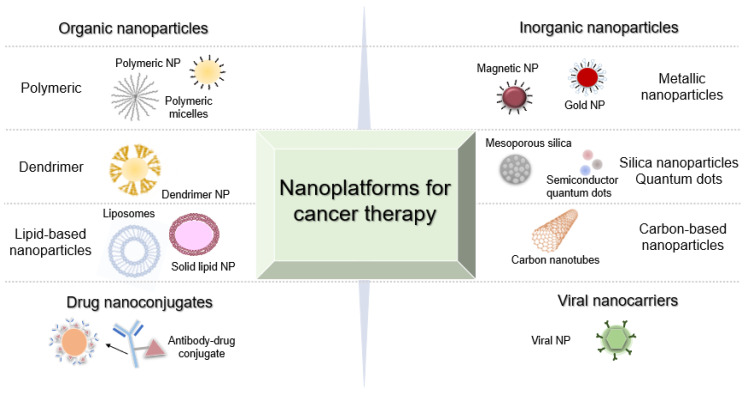
Schematic illustration of different nanoplatforms used for cancer diagnostics and therapy (e.g., organic and inorganic nanoparticles, drug nanoconjugates and viral nanocarriers).

For the past years, a variety of nanotechnology schemes, based on organic, inorganic, lipid, protein and synthetic polymers, have been employed in the development of new cancer therapies [49,50,51,52]. These nanoplatforms, which include liposomes, micelles, dendrimers, metallic, silica and polymer nanoparticles, may be synthesized in a multitude of chemical compositions and displaying a wide range of unique physical properties—See Figure 1 and Table 3 for a summary of these characteristics, referring to recent examples. One of the main interests of these approaches is the possibility to combine different therapeutic modalities, provided either by the fundamental properties of used nanomaterials or by the cargo, with the capability to visualize, in real-time, the distribution and accumulation at the target place in the body, which may be coupled to the chance of detecting critical biomarkers of disease (diagnostics) [49,52]. This way, nanotechnology enabled the surge of (nano)theranostic, providing therapy and diagnostics on a single platform.

Nanomedicines may be constructed to be selective in targeting cells and/or tissues via the incorporation of biomolecules to improve the uptake of therapeutic agents by cancer cells. Active and passive targeting may be used to direct therapeutic moieties into the desired cells and cell-targets. The former makes use of pathophysiologic alteration of the fluid/solutes’ dynamics in the body, while the later requires the use of particular biomolecules (e.g., antibodies, small molecules, peptides, etc.) that are recognized by the target cell, thus improving selectivity and uptake [49,52]. Nanomedicines also have allow to target multiple tumor markers simultaneously and deliver a wide range of (bio)chemotherapeutics, which may result in a synergistic approach to address cancer heterogeneity and resistance [49,50]. Nanomedicines may also extend circulation times of compounds and mediate stimuli-responsive drug release and uptake by cells [74,75].

### 2.1. Metal NPs for Gene Silencing

Several types of nanosized vehicles have been developed for the vectorization of TNAs, such as polymeric particles, dendrimers, semiconductor quantum dots, amino acids, liposomes, carbon-based nanostructures, viral vectors, silica and metallic nanoparticles [7,76]. These vehicles are essential to deliver TNAs directed against cancer cells, even though the delivery efficiency of these carriers to malignant cells in clinical applications remains a challenge [76,77,78]. Viral vectors usually demonstrate higher transfection efficiency than their non-viral counterparts, but tend to trigger strong immune responses by the host and usually display severe cytotoxicity [79]. The ease and low cost of production, together with reduced immunotoxicity of the non-viral vectors prompt for their use in substitution of traditional viral vectors [80]. Still, lipid nanoparticles (LNPs) is the most common nanoparticle-based approach for the delivery of TNAs [81,82].

Currently, metallic NPs for have been considered effective vectors for the delivery of RNA silencers, AuNPs take a leading role. Usually, these NPs are modified with different targeting agents or probes (e.g., addition of polyethylene glycol or PEG for improved biocompatibility, antibodies, TNAs) as active moieties for targeting cancer cells and optimizing retention within tumor. Also, when directing nanoscale structures at cancer sites, one may profit from the physiologic alterations within the tumor to passively target these sites–passive targeting. The paradigm is the enhanced permeability and retention (EPR) effect, which promotes the facilitated extravasation of NPs through the endothelia of surrounding vessels into the tumor. EPR occurs due to leaky endothelia resulting from the unstructured development and expansion of new vessels supplying the tumor with the greatly needed nutrients, which have grown too fast and without proper maturation to provide for fully structurally stable and finished architecture [7]. In the case of triggered targeting, NPs may release their content following a stimulus, such as a magnetic field (e.g., magnetic stimuli), an electric field, ultrasound or light (e.g., photothermy, optical hyperthermia) [83,84,85]. Metal nanoparticles, such as iron oxide (IONP), superparamagnetic iron oxide (SPION), silver (AgNP) and gold (AuNP), have been exhaustively used in several biomedical applications due to their physical and chemical properties, which are strongly dependent on size, shape, surface area, amphiphilicity and biocompatibility. Most of these NPs are often synthesized through simple procedures, and may be easily functionalized with a plethora of biomolecules, usually via simple chemistry (e.g., nucleic acids, proteins, drugs/compounds) to provide for biological activity and functionality [7,85,86].

Magnetic nanoparticles (MNPs) have mainly been used as contrast agents to enhance the resolution and accuracy in magnetic resonance imaging. Upon exposure to an external magnetic field, MNPs will provide for distinct relaxation times that increase imaging discrimination depending on the surrounding tissues, thus providing for additional resolution. Additionally, MNPs have also been used as nanocarriers, where the use of a magnetic field might result in accumulation in a particular spot in the organism. Furthermore, by using an alternating magnetic field these MNPs may act as “heat-converters”, assisting strategies relying on mild hyperthermia (42–45 °C), which have been shown to improve gene delivery efficiency through the cell compartments, enhancing gene silencing effects [87,88,89,90].

Nevertheless, perhaps the most used metallic nanoparticles for gene therapy have been based on gold. In fact, AuNPs have been described as specific and efficient carriers, used in target-specific delivery of RNAi (e.g., siRNA, miRNA, shRNA), alone or in combination with drugs or antibodies, for example [91,92]. In fact, AuNPs have been used as theranostic systems in cancer, due to their biocompatibility and unique physico-chemical properties, including their optical behavior derived from the localized surface plasmon resonance (SPR). The easiness of synthesis and of functionalization via thiol base chemistry make them appreciated vehicles to adsorb and/or graft TNAs to the surface for simplicity of delivery. It should be noted that most usage has been related to conceptual illustrations of the delivery potential exploiting the more traditional gene editing/modulation tools, primarily in vitro. Some of these concepts have also been evaluated in ex vivo and in vivo, and only but a few really making their way into the clinical setting.

For example, spherical AuNPs are good vectors for gene therapy, since they are easy to synthesize and usually exhibit low cytotoxicity, large specific surface areas that are easy to functionalize, high cell uptake and fast endosomal escape, making them biocompatible [7,29]. Due to some of the NPs’ properties (surface charge, polarity, etc.), cellular uptake occurs through active transport, mostly by endocytosis through any of the available pathways, e.g., clathrin-mediated, caveolae-mediated or clathrin- and caveolae-independent endocytosis. Endosomal escape is one of the most challenging barriers of NP drug and gene delivery since it affects transfection efficiency. Depending on the type of NP and functionalization of the surface there are several ways by which the AuNPs escape the endosome (before or after release the cargo), but in most cases this is thought to occur via the “proton sponge” effect [93,94]. Interestingly, AuNP as carriers have focused on more standard RNAi tools, such as ASOs and siRNAs, with growing applications related to CRISPR-Cas9, whereas vectorization of TALENs, ZFNs and meganucleases have lagged behind. This might be due to the fact that the latter molecular actuators are themselves less notorious and/or because the use of nanoscale vehicles are still not so widespread, even considering the reports on dendrimers and liposomes delivering strategies—See Table 4.

The delivery of TNAs to cancer cells using spherical AuNPs has been widely described in literature, not only to tackle and destroy malignant cells but also as means to modulate the TME [92,95]. The major advantage of spherical gold nanoconjugates is the selective delivery of TNAs to the nucleus, avoiding degradation by nucleases. However, aggregation of these nanoconjugates after binding with oligonucleotides is a common constraint to their usage [7,96]. Once these AuNPs are introduced into the organism, which may occur via direct intravenous administration, instillation, enteric and/or a range of implantable matrices for controlled systemic delivery, two main events tend to occur-aggregation of NPs and reaction with proteins (i.e., formation of protein corona) [97,98]. Depending on the AuNPs’ size, surface chemistry and morphology, proteins can mediate the formation of a corona on the nanoparticles’ external surface, prompting recognition by cells, which can be translated in improved or decreased colloidal biostability, improved of deferred cell uptake, recognition by the immune response (e.g., macrophage activation). As such, besides the cargo and targeting moiety, there is usually the need to graft additional biocompatible, hydrophilic molecules to the NPs so as to evade protein corona formation. Colloidal stability is also an issue regarding naked AuNPs in biological environments, since aggregation can be induced via medium components, serum proteins or by the formation of protein corona, strongly influencing AuNP-cell interactions, effective concentration and cellular uptake [99]. Also, a recent review reveals that most nanocarrier systems tend to be trapped in the liver, spleen and kidney, and only 0.7% (median) of the administered dose is delivered to the solid tumor [100]. The choice of surface chemistry is critical for the loading capability and to overcome the referred bottlenecks, passively directing the carriers towards the target site and to govern circulation half-life, to modulate cell uptake and evade or trigger the immune system. What is more, surface chemistry plays a critical role in those time and spatial controlled released systems that allow the cargo to be deployed in a “smart” way.

Different types and shapes of gold nanoparticles have been used in a range of smart designs to convey TNAs for cells and tissues, but mostly relying on the use of silencing approaches of canonical gene targets via siRNA and/or ASOs (as exemplified in Table 4). In fact, there is a wide range of imaginative ways AuNPs may be used to carry nucleic acid moieties, whose impact might be enhanced via combination with different molecular actuators for gene modulation, such as those exemplified in Table 2.

#### AuNPs for Nucleic Acid Delivery

**Figure 2:**
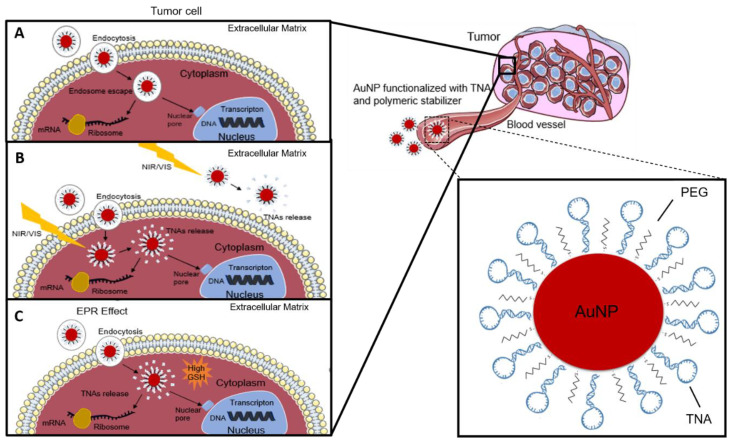
Functionalized spherical gold nanoparticle (AuNP) for effective delivery of gene silencers into tumor cells. (**A**) Via passive/active targeting and cell uptake. (**B**) Light-regulated release with spatial and temporal control (outside or inside target cell). (**C**) Intracellular release via chemical reduction of bonds, e.g., GSH-mediated reduction of thiol bond. EPR effect: enhanced permeability and retention effect; GSH: glutathione; NIR/VIS: Near Infra-Red/Visible PEG: polyethylene glycol; TNA: therapeutic nucleic acid, such as antisense DNA and RNAi therapeutic (e.g., siRNA, shRNA, miRNA).

Several functionalization strategies of spherical AuNPs for nucleic acid delivery have been described. Due to their surface properties and ease of functionalization, AuNPs used for the delivery of TNAs are usually conjugated with assorted molecules in order to ensure improved efficiency of delivery and uptake by target-cells, to avoid enzymatic breakdown and evade triggering an immune response that would remove these conjugates from the organism. To improve stability, some of these molecules are adsorbed to the AuNPs’ surface either by covalent bonding (gold-thiol bonding is the most common) or by electrostatic interaction [120]. Stabilization techniques include the use of nucleotide monolayers, making TNAs less susceptible to degradation by nucleases, while simultaneously decreasing toxicity, for example by the use of cationic polymers, such as PEI [121,122], that exhibit higher stability in physiological environments and transfection efficiency; cationic lipids, that allow the formation of lipoplexes and increased stability of lipid-DNA complexes [123,124]. PEG is one of the most used stabilizers for AuNP to permit TNA delivery, due to better stability against enzymatic/non-enzymatic hydrolysis, decreased acute cell toxicity, and increased circulation time [125,126]. For gene silencing applications, chemical functionalization with polymeric stabilizers, such as PEG, polyethyleneimine (PEI) and polyglycerol (PG), confer neutral charge surface and are essential to increase biocompatibility, solubility, and circulation time in biological systems. Besides that, neutralization of charged nanoparticles assures low adsorption to circulating plasma proteins (e.g., opsonins), avoiding AuNP elimination from circulation through phagocytosis. Nucleic acids with their evident negative charge may bind to cationic AuNPs via ionic interactions [7,127]. AuNPs can also be functionalized with thiolated oligonucleotides, alkythiol-terminated oligonucleotides, amine terminated siRNAs or cysteamine-terminated miRNAs [76]. Likewise, the AuNPs’ affinity to bind oligonucleotides or even biomolecules containing thiol groups makes them appropriate vehicles for siRNA delivery—See Figure 2. These systems have been used in a variety of gene silencing applications and tracing specific gene targets in nanotheranostics.

AuNP mediated delivery of TNAs is highly influenced by the design and assembly of the nucleic acid into functional nanoconjugates. Amongst the range of conjugation strategies onto AuNPs, covalent attachment and supramolecular assembly of TNAs has been the preferred approach in gene silencing applications, mainly because it relies on the simple and straightforward reaction via interaction between Sulphur present in thiolated (modified) TNAs and the surface of AuNPs (S-Au binding). Non-covalent conjugates are also appealing alternatives, since supramolecular assembly enables the usage of unmodified nucleic acids, while retaining the efficacy in gene therapy applications [94,119,128,129]. Other options for TNA loading include mixed-monolayer-protected AuNPs (MM-AuNPs) [130], amino acid-functionalized AuNPs (AA–AuNPs) [131], and layer-by-layer-fabricated AuNPs (LbL-AuNPs) [132], etc. LbL-AuNP synthesis using negatively charged TNAs and positively charged AuNPs render strategies for the controlled release of nucleic acids, depending on the composition and arrangement of the multi-layer constituents [133].

Controlled and stimuli-responsive release of TNAs can also be achieved through different processes. Most of these strategies rely on the extracellular matrix conditions, such as pH, temperature, ionic strength, enzymatic activity or intracellular reactivity, such as those mediated by intracellular reduction of the thiol bond (e.g., via glutathione, GSH). For example, DeGSH exhibits an overall negative charge and can act as a reducing agent, by interacting with the cationic surface of AuNPs and reversing particle charge, which results in ligand (TNA) release [134]. GSH is the most abundant thiol species in the cytoplasm, with intracellular concentrations significantly higher than extracellular levels, providing a mechanism for selective intracellular release.

Additionally, several concepts have been put forward relying on the application of external stimuli, such as irradiation with a suitable wavelength, ultrasounds, etc., which might provide for spatiotemporal control of nucleic acid release. For example, several studies have described the design of photolabile AuNPs, whose function was to release DNA in supramolecular AuNP complexes through irradiation [135]. By synthetizing positively charged AuNPs bearing a photocleavable o-nitrobenzyl ester linkage, the global surface charge of AuNPs could be reversed under irradiation, yielding a negatively charged carboxylate group and successively releasing negatively charged DNA by electrostatic repulsion.

AuNPs have been characterized as efficient, specific, and selective vehicles for gene silencing and genome editing tools. CRISPR/CAS9 system is a great approach for the simultaneous edition of multiple loci, but have some limitations, such as cleavage at off-target sites [40,136]. Recently, Shahbazi et al. reported an efficacious nanoformulation with low toxicity towards the delivery of CRISPR tools based on colloidal AuNPs. These new vectors were compared to electroporation and virus nanocarriers for ex vivo gene editing in hematopoietic stem and/or progenitor cells and showed far less direct cell toxicity. These AuNP formulations combined with different CRISPR nucleases at multiple *loci* is easily up taken to the nucleus, exhibiting high specificity to target cells [137]. Another good example was proposed by Wang et al. who described a multifunctional gold nanocarrier for effective delivery of Cas9-sg-*Plk-1* plasmid into melanoma cells. This gold nanoconjugate coated with lipids was capable to release the cargo by a trigger into the cells, promoting the downregulation of *Plk-1* and inhibit the proliferation of tumor cells in vitro and in vivo [70]. Multifunctional AuNPs combined with CRIPR/Cas9 genome editing provide an adaptable method for treatment of several conditions, such as cancer and other conditions with clear genetic background.

The capability to control the amount of cargo to be conveyed by a nanocarrier one critical aspect impacting the therapeutic efficacy. For example, it has been proposed that the programmed assembly of nucleoprotein nanoparticles (NNPs) conjugating DNA and ZFNs for the targeted delivery of therapeutically relevant proteins, where dsDNA with multiple zin-fingers (for specific binding) is conjugated to inorganic nanoparticles, which could be used to improve efficacy. This approach enabled controlled stacking of proteins on the NNPs, providing for a uniform coat with high stability, high efficiency of intracellular delivery and enhanced specificity [40,138]. Several other techniques have been used to modify RNA with riboswitches, ribozymes, or endonucleases. For example, Petree et al. developed the first concept for a splicing nanoparticle system for gene editing with potential for in vivo applications. This nanozyme system is based on AuNPs functionalized with three different enzymes, capable of cleavage and ligate RNA targets and splicing RNA stem-loops. However, the efficiency of the nanozyme must be increased to allow to transiently edit genes and for in vivo splicing [139].

Several in vitro and in vivo studies data highlight the powerful capability of delivery of gene silencers using AuNPs. Conde et al. used an antisense gold nanobeacon for gene silencing in colorectal cancer cells transfected with *EGFP* plasmid, proving the efficacy and non-toxicity of the silencer while providing means to simultaneously evaluate the gene expression levels [91]. In another example, multifunctional AuNPs have been engineered to deliver siRNA directly targeted at malignant cells in a mouse lung cancer model, which was capable to down-regulate *cMYC* oncogene, leading to a suppression of cell proliferation and improved survival [140]. Another example of effective gene silencing was put forward by Vinhas et al. that combined gene silencing via a AuNP loaded with an ASO targeting *BCR-ABL1* and imatinib, enhancing cell death in chronic myeloid leukemia (CML) cells [141]. Another interesting example, but targeting acute myeloid leukemia (AML), has been demonstrated though the administration of AuNPs functionalized with a nuclear localization signal, and designed to target miR-211 for inhibition and hamper nucleolin function in AML cells. In vitro testing showed effective silencing of the NCL/miR-211/NFkB/DNMT1 pathway, leading to the synergistic arrest of AML cell proliferation; corroborated by the in vivo testing with extension of AML mice survival with disease regression [142].

AuNPs have also been proposed as a more universal carrier for delivery of shRNAs used against cancer [143]. The in vitro studies performed by Ryou et al. consisted in the synthesis of shRNA specific to *p53* and *Mcl-1* gene combined with AuNPs, showing the transfection efficiency into human embryonic kidney cells and cervical carcinoma cells lines. In short, gold nanoconjugates for shRNA delivery have shown higher efficacy when compared to liposome-based shRNA delivery methods [45,144,145]. Interestingly, in cancer therapy, synergistic combinations have been developed to achieve higher efficacy. What is more, the antitumor use of self-assembled RNAi-AuNP nanoconstructs that combine the gene silencing capability with directed photothermal ablation of the tumor mediated by the same AuNPs has also been proposed. Mild photothermy has been shown to increase cell membrane fluidity, facilitating cellular uptake, and endosomal escape. Also, TNA release can be triggered through the application of laser pulses of strong intensity, improving gene delivery [146,147]. The capability of gold nanoconjugates to decrease vesicle trafficking (e.g., exosomes) in breast cancer cells via silencing of RAB27A, a crucial gene in exosome trafficking pathway, has also been shown [148].

Still, these examples still require long-term toxicological studies to assess the real impact of these gene silencing vectors in biological systems.

### 2.2. Translation to the Clinic

Nanomedicines continue to evolve rapidly, particularly on what new drug-delivery strategies, innovative treatment modalities are concerned. Still, clinical translation of these nanoplatforms is not as fast as desired. What is more, there have been several reports of repurposing of old drugs but in combination with these innovative aspects of nanomedicines, thus providing a new life to some of these compounds. AuNPs’ particular properties provide for additional properties and useful utensils to be applied in stimuli-responsive functions for localized and timely release of cargo, for the thermal heating directed at the ablation of malignant cells and the possibility of high accuracy imaging modalities (e.g., molecular computerized tomography) [149,150,151]. Multifunctional AuNPs carrying TNAs and/or gene editing molecular tools have been mostly evaluated in in vitro models, but several of these concepts have also been proven in vivo. However, it must be said that most of these concepts do not make into the systemic evaluation required for further clinical development and translation, mostly due to hurdles relating to scale-up of production and precise physical characterization of these nanoconjugates, and for the complexity of assessing chronic and whole organism’s nanotoxicology [152,153].

Still, even though clinical trials focused on the sue of AuNPs for gene silencing strategies remain infrequent, some of the hurdles preventing their smooth translation are starting to be overcome. For example, the drug NU-0129 consists spherical AuNPs whose surface has been functionalized with nucleic acids targeting *BCL2L12*, to prevent tumor growth in glioblastoma/gliosarcoma (NCT03020017; Phase 1; Active) [154]. Regardless of these advances in precision nanomedicine, which allows for a more personalized and efficient treatment of cancer, there are still significant challenges facing effective translation of nanomedicine into clinics. Some of the hurdles refer to the scalability issues of nanoparticle production, batch-to-batch reproducibility, and incomplete information regarding the interactions between biological system and nanostructure due to the shear complexity of systems and biological pathways. What is more, these nanomedicines directed at gene modulation still require an effective increase of therapeutic indexes, which is met with reluctance by the biopharma industry to invest further in this new but rather unproven field. Additionally, there are several issues relating to the regulatory framework and safety guidelines from authorities and agencies [155,156]. Some experts in the field consider that distinctive strategies should be considered in the design of preclinical studies so as to potentiate translation to the clinics [157]. Since most tools and approaches in nanomedicine in gene silencing, including those aspects related to synthesis and precise characterization, are extremely new, they still require consolidation of practices, deeper studies of their biological effect, and combination of data arising from cell and animal models [7,157].

## 3. Conclusions and Future Perspectives

Despite the tremendous development in the field, current cancer treatment options are still mostly based on invasive and non-specific strategies to tackle the growth of malignant cells and disrupt their progression. Most of the therapeutic strategies rely on the use of chemotherapeutics that lack that desirable molecular and cell selectivity to spare healthy cells and tissues when killing the tumor. Novel target-specific approaches aim at the selective delivery of the therapeutic moiety straight to the cancer cells based on their specific molecular profile. This molecular profile has also become a target of choice for gene therapy, for which genome editing tools and RNAi approaches have taken the lead, putting forward promising new molecular modulators that are capable to correct malignancy at the molecular level. Thus far, perhaps the biggest hurdle for the effective use of these gene therapy approaches has been the capability to deliver the TNA to the target tissue. Nanomedicine, and nanoparticles in particular, have been providing for innovative platforms to overcome these bottlenecks and doing so in combination with a range of physic-chemical properties that potentiate the probability of therapeutic success. In fact, not only these nanoparticles provide for effective vectorization platforms for TNAs, but they may also act as imaging and therapeutic moieties themselves, paving the way for efficient tools in (nano)theranostic. Some of these have been combined in ingenious strategies for the simultaneous delivery of TNAs and cell disruption mechanisms that provide additional cell death and/or molecular imaging protocols.

Molecular nanomedicine applied to cancer therapy relies mainly on the precise delivery of RNAi therapeutics, drugs, or antibodies into cancer cells. Ideally, nanoconjugates are vectorized into cells with maximal transfection efficiency in selected target cells and low toxicity to neighboring healthy cells and tissues. The use of multifunctional AuNPs to deliver gene modulators and silencers into cells has been pushing forward as an alternative to conventional transfection agents with several advantages in terms of biodistribution and, above all, combination with other therapeutic and imaging modalities provided by the nanoparticles themselves. These nanoparticles are easily functionalized with TNAs (e.g., siRNA, miRNA, shRNA) and with biomolecules capable to direct these nanocarriers to the target cells (e.g., antibodies). Perhaps because of the intrinsic properties of the metal, AuNPs have been prominent in these efforts. Despite the range of available molecular tools and TNAs capable to deliver a therapeutic response as discussed above, it is interesting to note that the majority of systems relying on AuNPs for delivery purposes focus on siRNA and ASOs as the real “active principles”. Still there has been increasing interest directed towards CRISPR-Cas9 systems. Still, in principle reports on several concepts have highlighted that all these individual systems might be combined, and their efficacy compared so as to deliver optimized platforms. Interestingly, often the more simplistic approaches render the optimal effect and do so with less complex processes, which is a key aspect impacting industrial development and clinical translation.

Over the last years, multifunctional AuNPs for selective gene delivery to cancer cells have mainly been studied in in vitro cell models and in a few very particular in vivo animal models. In fact, AuNPs for gene therapy applications still face great challenges to prove their efficacy in more elaborate animal models that is a pre-requisite to move to the clinics. These AuNP must progress beyond their current proof-of-concept statute, which requires extensive testing and characterization before translation into clinical trials. Perhaps two of the most urgent requirements for effective translation of these gene therapy systems based on nanotechnology are the scale up of production and capability for characterization with reproducible precision, and the extensive studies of toxicity in the organisms, which still require a consensual protocol of assessment that suit the nanomaterials in hand.

Multifunctional nanoplatforms, such as AuNPs-siRNA combined with drugs or antibodies, show plenty of potential advantages for combinatory cancer therapy, improving the efficacy and enhance specific gene delivery. Furthermore, triggered targeting based on an external stimulus (e.g., photoinduced delivery, magnetic) that may be combined with photothermal ablation (or enhancement) is slowly proving to be proficient in in vitro and in vivo studies for the improvement to siRNA-based therapy.

## Figures and Tables

**Table 1:** Overview of the use of gene editing tools in clinical trials.

Platform	Condition/Disease	Target	Cells	Vectorization	Clinical Trial Reference (Phase)
ZFN	HIV-1 infection	CCR5	T (CD4+)	Adenovirus	NCT01252641 (I/II)
			T CD4/CD8	mRNA	NCT02225665 (I/II)
			HSPC (CD34+)	mRNA	NCT02500849 (I)
	HPV-induced precancerous Lesions of cervix	HPV16/18 E7	Epithelial	DNA	NCT02800369 (I)
	Hemophilia B	Factor IX gene	Hepatocytes	AAV	NCT02695160 (I)
TALEN	Relapsed/refractory B-ALL	CD52, TRAC	CAR T	Lentivirus	NCT02808442 (I)
	HPV-related cervical cancer	HPV16/18 E6/E7	Epithelial	Plasmid	NCT03226470 (I)
TALENs coupled to CRISPR/Cas9	HPV-associated cervical cancer	HPV16/18 E6/E7	Epithelial	Plasmid	NCT03057912 (I)
CRISPR/Cas9	AML	CD123, TRAC	CAR T	mRNA	NCT03190278 (I)
	Various cancers	PDCD1	T	DNA	NCT02793856 (I) NCT03081715 (I)
	Relapsed/refractory CD19+ leukemia and lymphoma	TRAC, B2M	CAR T	Lentivirus, electroporation	NCT03166878 (I/II)
		CD19 and CD20 or CD22, TRAC	CAR T	Lentivirus, electroporation	NCT03398967 (I/II)
	T cell leukemia, lymphoma	CD7, CD28	CAR T	-	NCT03690011 (I)
	β-thalassemia	BCL11A	HSPC (CD34+)	-	NCT03655678 (I/II)
	Sickle cell disease	BCL11A	HSPC (CD34+)	-	NCT03745287 (I/II)

AAV: adeno-associated virus; AML: acute myeloid leukemia; BCL11A: mouse B cell lymphoma factor 11A; CAR T: Chimeric Antigen Receptor T; CCR5: chemokine receptor 5; CRISPR: clustered regularly interspaced short palindromic repeat; HPV: Human papillomavirus; HSPCs: hematopoietic stem/progenitor cells; PDCD1: programed cell death 1; TALEN: transcription activator-like effector nuclease; TRAC: T cell receptor alpha chain; ZFN: zinc-finger nuclease.

**Table 2:** Description of gene therapy tools applied to cancer treatment.

Gene Therapy Tools	Characteristics	Advantages	Limitations	Application in Cancer Therapy	Ref.
CRISPR/Cas9	CRISPR/Cas9 facilitate efficient multiplex genome editing, with the possibility of simultaneous deletion or insertion of multiple DNA sequences.	CRISPR/Cas9 is fast to develop, simple and cost-saving in comparison with other gene editing tools. Potential for simultaneous multiple loci editing.	High frequency of random integration. Activation of non-homologous end joining DNA repair pathway (may lead to microdeletions at the DSB site).	T-Cell modification in cancer therapy.	[15,16,40,41,42]
ZFNs	DNA-binding domains recognize trinucleotide DNA sequences (longer DNA sequences may also be targeted).	ZFN may be designed to include a variety of effector domains to recognize virtually any DNA sequence.	Off-targets effects are high. Expensive and hard to use technique.	Clinical application other than cancer (HIV).	[21,43]
TALENs	DNA targeting specificity comes from the fused bacterial TALE proteins. TALEN arrays recognize only a single nucleotide (as opposed to ZFNs) and it has no impact on the binding specificity.	TALEN engineered nucleases show better specificity and efficiency than ZFN.	The major limitation is the cloning of the large modules in series and join these modules in designated order by ligase in an efficient way.	Clinical trials -Cervical intraepithelial neoplasia; hematological malignancies.	[9,40,43]
RNA interference	RNAi generally used to down-regulate gene expression. Also, the effector molecules (e.g., siRNA) may be vectorized alone or expressed from suitable plasmids.	RNAi uses the cells’ machinery, facilitated by short interfering RNA molecules.	Short half-lives due to RNases. Low biochemical stability. High costs.	Silencing oncogenes in several cancer–clinical use.	[31,44,45,46]
Antisense	AON are small DNA or RNA molecules complementary to their target mRNA. Binding to their target result in alteration of mRNA splicing or degradation of target transcripts.	Simple to design and vectorize inside the cell	Obstacles for in vivo delivery (immunogenic) that may cause side effects. Short half-lives due to RNase activity.	Clinical application other than cancer (e.g., thalassemia).	[29,31,47,48]
Meganucleases	Meganuclease technology involves re-engineering the DNA-binding specificity that naturally occurs in the family of homing endonucleases.	Meganucleases are the smallest class of engineered nucleases, making them potentially amenable to all standard gene delivery methods since they offer fewer off-targets effects.	Meganucleases are difficult to construct, time-consuming and high costing limiting their use as gene editing tools.	No reports on clinical applications.	[27,43]

**Table 3:** Type of nanoparticles (NPs) used as nanocarriers in cancer therapy.

Type of NPs	Characteristics	Application	Limitations	Examples	Gene Editing Tool	Ref.
Dendrimer	Small (1–15 nm) branched polymeric NPs High water solubility, high cargo capacity.	Targeting cancer cells and injured tissues	Synthesis is quite time-consuming; Some toxicity issues in drug incorporation and release.	PAMAM dendrimers used as drug delivery systems.	Boronic acid-rich dendrimer as vector for CRISPR/Cas9. Meganucleases	[53,54,55,56]
Liposome	Nanostructures made of amphiphilic molecules (polymers, lipids) with good biocompatibility and cell uptake.	Delivery of hydrophilic or hydrophobic cargo depending on lipid constitution.	Poor stability, rapid degradation and clearance. May trigger lipid oxidation (long-term).	Doxorubicin in a heat sensitive liposomal formulation-ThermoDox^®^ (Celsion) in phase III clinical trial in primary hepatocellular carcinoma; in phase II for refractory breast cancer and colorectal liver metastasis.	Lipid delivery systems for siRNA delivery. Meganucleases	[56,57,58,59]
Polymeric NP	Natural (proteins and polypeptides) or synthetic. Biocompatibility and biodegradable.	Controlled drug release, protection of drug molecules specific targeting.	Low transfection efficiency Some cytotoxicity.	Most used is PEI (Polyethyleneimine) in nanosized ionic complexes (polyplexes).	Delivery of plasmid DNA (CRISPR–Cas9) in PLGA.	[60,61,62]
Carbon NP	Carbon dots, graphene, oxides, and carbon nanotubes (CNT). Unique mechanical and optical properties.	Imaging and drug delivery applications.	Cytotoxicity.	Stimuli responsive drug delivery systems.	siRNA delivery and intracellular tracking of siRNA (nanotheranostics).	[63,64,65]
Quantum Dots	QDs are luminescent nanoprobes that present high photostability, i.e., no photobleaching.	Used in imaging, detection and targeting.	High toxicity due to the CdSe (metallic core of the NP).	Quantum Dots can act as photosensitizers, producing reactive oxygen species (ROS) upon light irradiation.	No reports on clinical applications.	[66,67]
Gold NP (AuNP)	Metallic core NPs with unique optical and physical-chemical properties.	Primarily used for labelling applications; may be used in theranostic tools.	Cytotoxicity, biodistribution, retention time, and physiological response of NPs.	AuNP can be used as photothermal agents in hyperthermia, and nanocarriers for gene silencing.	Deliver of CRISPR-Cas9 with/without external stimuli	[39,68,69,70]
Magnetic NP (MNP)	Actuated by an external magnetic field.	Imaging.	Cytotoxicity of ion core.	Ferucarbotran (Resovist^®^)-clinically approved superparamagnetic iron oxide nanoparticles (SPIONs) coated with carboxydextran for the enhancement of MRI contrast of the liver.	CRISPR/Cas9-PEI-MNP.	[71,72,73]

**Table 4:** Most common type of AuNPs used in cancer therapy.

AuNPs	Characteristics	Advantages	Limitations	Applications	TNAs	In Vivo Distribution	Ref.
AuNRs	Elongated NPs, showing longitudinal plasmon wavelength with nearly linear dependence on their aspect ratio.	Tunable properties, including SPR.	Low drug loading capacity. Poor control over size distribution.	Tunable optical resonance in the NIR for in vivo applications, such as imaging, photothermy.	siRNA DNA aptamers ASOs RNA decoys	Accumulation in the liver, long circulation time, and high accumulation in the tumors.	[101,102,103,104,105,106,107,108]
AuNCus	Hollow with ultrathin and porous walls. Easy to synthesize (scale up).	Tunable sizes and scalability.	Few data about toxicity, biodistribution, and physiological response.	Theranostics (SPR can be tuned between 600–1200 nm); hollow interiors allow encapsulation; porous walls for ease drug release.	siRNAs miRNAs	Medium level accumulation in the liver, kidneys and spleen. Rapidly excreted.	[102,106,109,110,111]
AuNShs	Spherical with a dielectric core covered by a thin gold shell.	Improve in vivo bioavailability and controlled drug release.	Lack of targeting efficacy. Limited tracking/monitoring in vivo.	Controlled/triggered drug delivery via irradiation (NIR).	siRNA ASOs	Short circulation times, accumulation in liver and spleen. No induction of tissue damage (necrosis, inflammatory infiltrate or fibrosis) liver, spleen, kidney or bone marrow.	[112,113,114,115,116]
AuNSs	Spherical solid.	Ease of functionalization. Enhanced cellular uptake.	Prone to aggregation. Multitude of possible cell uptake routes render difficult to control.	Extremely versatile for photothermy, and combined therapy.	ASOs siRNAs miRNA	Short circulation time and accumulation in the liver with low accumulation in the tumors.	[91,117,118,119]
